# Risk Factors of Influenza-Associated Respiratory Illnesses Reported to a Sentinel Hospital of Lahore, Pakistan: 2015-2016

**DOI:** 10.1155/2021/2460553

**Published:** 2021-10-27

**Authors:** Saima Hasan, Muhammad Iqbal, Richard J. Webby, Jennifer DeBeauchamp, Hamad Bin Rashid, Mansur-ud-Din Ahmad, Jawad Nazir, Shakera Sadiq, Abdul Waheed Khan, Mamoona Chaudhry

**Affiliations:** ^1^Department of Epidemiology and Public Health, University of Veterinary and Animal Sciences, Abdul Qadir Jilani Road, Lahore, Pakistan; ^2^World Health Organization Collaborating Center for Studies on the Ecology of Influenza in Animals and Birds, Department of Infectious Diseases, St. Jude Children's Research Hospital, 262 Danny Thomas Pl, Memphis, TN 38105, USA; ^3^Department of Surgery and Pet Sciences, University of Veterinary and Animal Sciences, Abdul Qadir Jilani Road, Lahore, Pakistan; ^4^Department of Pathobiology, Riphah College of Veterinary Sciences, Riphah International University, 12 Kilometer Raiwind Road, Lahore, Pakistan; ^5^Department of Microbiology, University of Veterinary and Animal Sciences, Abdul Qadir Jilani Road, Lahore, Pakistan; ^6^Virology Laboratory, Treidlia Biovet, 76/45 Powers Rd, Seven Hills, NSW 2147, Australia; ^7^Medical Education & Community Medicine, Poonch Medical College, Diwan Chowk, Rawalakot, Azad Jammu and Kashmir, Pakistan

## Abstract

Epidemiological data about determinants of influenza A virus (IAV) in the Pakistani population is scarce. We aimed to conduct a prospective hospital-based active surveillance study from October 2015 to May 2016 to identify potential risk factors associated with IAV infection among patients with influenza-like illness (ILI) and severe acute respiratory illness (SARI). Surveillance was conducted in Lahore General Hospital, selected as a sentinel site in Lahore District, Pakistan. Nasal/throat samples were collected along with epidemiological and clinical data from enrolled patients. Real-time reverse-transcription polymerase chain reaction (rRT-PCR) was performed to identify IAV and its subtypes (H1N1pdm09, H3N2). Data were analyzed to determine risk factors and risk markers associated with IAV infections. A total of 311 suspected ILI and SARI cases were enrolled in the study, and among these 50 were IAV-positive. Of these 50 confirmed cases of IAV, 14 were subtyped as H1N1pdm09 and 15 were H3N2; the remaining 21 were untyped. A final multivariable model identified four independent risk factors/markers for IAV infection: exposure history to ILI patients within last 7 days and gender being male were identified as risk factors of IAV infection, while use of antibiotics prior to hospital consultation and presence of fever were identified as risk markers. We concluded that adopting nonpharmaceutical interventions like hand hygiene, masks, social distancing, and where possible, avoiding identified risk factors could decrease the risk of IAV infection and may prevent imminent outbreaks of IAV in the community.

## 1. Introduction

Influenza is a highly infectious, mild to severe respiratory disease of birds, humans, and other animals that is caused by influenza viruses. According to recent estimates of the World Health Organization (WHO), influenza A viruses (IAVs) cause recurrent regional epidemics leading to 3–5 million cases of severe respiratory illness and about 290,000 to 650,000 respiratory deaths worldwide [1]. Although the WHO report revealed 18,631 laboratory-confirmed deaths during the H1N1 pandemic in 2009, the statistical modeling showed a 10-time higher mortality rate using H1N1 mortality data from different countries [2]. The WHO stressed the importance of routine influenza surveillance incorporating clinical and epidemiologic data collection for risk factor identification as critical components of a comprehensive strategy in addition to laboratory diagnosis of various influenza subtypes [3]. Moreover, modifications in influenza vaccine strategies from 2019 in response to influenza epidemics by various subtypes highlighted the clear stratification of risk factors, subsequently improving the understanding of clinical management of severe disease [4]. IAV may cause high morbidity and mortality, with increased hospital expenditures and absenteeism from duties, hence decreasing productivity, which can burden a country's population and economy [5]. Data is scarce about the risk factors of influenza-associated morbidity and mortality globally and in Pakistan [6–8]. Evidence-based identification of risk factors associated with increased risk of influenza-associated morbidity and mortality in Pakistan could be used to target high-risk groups for interventions and to mitigate the spread of the disease. We conducted a hospital-based sentinel surveillance study and aimed to assess the relationship of various risk factors and risk markers with IAV infection in Lahore District, Pakistan, from October 2015 to May 2016.

## 2. Materials and Methods

Prospective hospital-based active surveillance was conducted in Lahore General Hospital (LGH), which is a major tertiary-level public sector hospital affiliated with Postgraduate Medical Institute (PGMI), Lahore. LGH was selected as a sentinel site for influenza surveillance due to its referral status and high patient load from the community within and at the periphery of Lahore District. Lahore is the second largest city in Pakistan after Karachi. According to the 2017 census, the human population of Lahore is 11,119,985 [9]. It is located geographically at 31°32ʹ59″ N 74°20'37″ E, has a semiarid climate, and experiences all four seasons (i.e., summer, winter, autumn, and spring) [10].

### 2.1. Study Population and Data Collection Procedure

**Figure 1:**
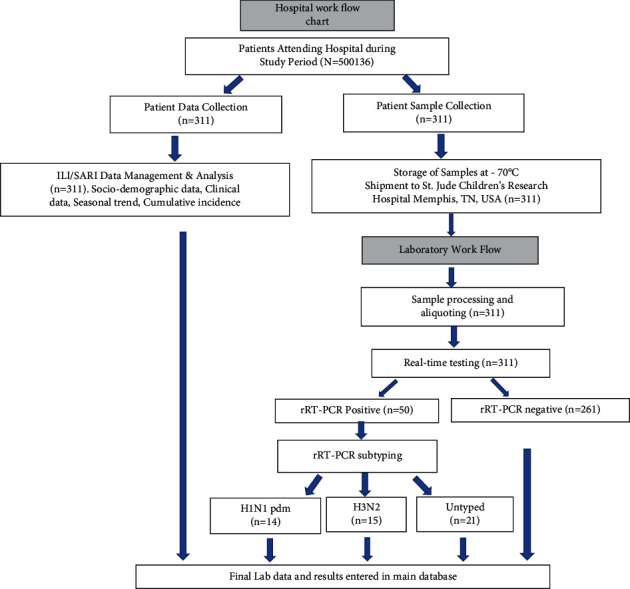
Flow chart of hospital surveillance activity.

The study population included all patients with respiratory illnesses (*N* = 513,126) coming from the catchment areas of LGH and attending outpatient or inpatient departments of the hospital, regardless of their age and sex. Eligible patients (*n* = 311) were screened by trained hospital staff according to revised WHO-prescribed case definitions of ILI or SARI and were enrolled in the study [11, 12]. A structured questionnaire about potential risk factors and risk markers for influenza A was used with both open-ended and closed-ended questions to collect epidemiological and clinical data during a face-to-face interview by trained health professionals. The questions were written in English but were asked in the local language (Urdu), which was better understood by participants. Risk factors for human infection with seasonal influenza were selected after extensive literature review, including WHO guidelines [11, 13–16]. The risk factors included in the questionnaire were sociodemographic information (name, address, age, gender, marital status, type of family, education, occupation, income per month), comorbidities, exposure status (to ILI cases, to poultry, etc.), vaccination status, and travel history to and from epidemic areas. The risk markers included were clinical characteristics (fever, cough, sore throat, sneezing, headache, etc.), use of antibiotics, etc. A unique, coded ID number was listed on each questionnaire and corresponding sample-containing tube to protect each participant's identity. The study started in October 2015 and ended in May 2016.  Figure 1 shows a flow chart of hospital surveillance activity.

### 2.2. Laboratory Diagnosis

A trained clinician at a sentinel site collected nasal and/or throat swabs from study participants who gave informed consent. The samples were placed in the viral transport medium as described by the WHO [17], transported in cool boxes at 4°C, and aliquoted in a BSL-2 (biosafety level-2) safety cabinet in the Disease Surveillance Laboratory, Department of Epidemiology & Public Health, University of Veterinary and Animal Sciences, Lahore, Pakistan. Sample aliquots were stored in a freezer at or below −70°C until they were shipped on dry ice to St. Jude Children's Research Hospital, Memphis, Tennessee, USA, for laboratory analysis by real-time RT-PCR. Viral RNA was extracted from 200 *μ*L of clinical sample by using RNeasy Mini Kit (Qiagen, Hilden, Germany) according to the manufacturer's instructions. Sample extracts were amplified by AgPath-ID™ One-Step rRT-PCR Kit (Applied Biosystems, Thermo Fisher, USA) for use with the 7500 Fast Real-Time RT-PCR system to detect M gene [18]. Influenza A subtypes H3N2 and H1N1pdm09 were further identified by using specific primers and probes as described by the Centers for Disease Control and Prevention (Atlanta, GA, USA) [19].

### 2.3. Statistical Analysis

Data sets were entered into EpiData software (version 3.1, Odense, Denmark), validated for errors and inconsistencies by random checking of digital data with the hard copy record, and then exported to Microsoft Excel (version 2013, Microsoft Office, USA) for further processing. All statistical analyses were conducted in R software (version 3.6.1, R Foundation for Statistical Computing, Vienna, Austria). The chi-square test was performed to test independence between two categorical variables. The set of variables were tested for collinearity by Spearman rank correlation. Univariable logistic regression analysis was applied to test the association of independent risk factors/markers with desired outcome (influenza A-positive and negative cases). Variables that fulfilled the selection criteria (i.e., *p* ≤ 0.25) qualified for further analysis by multivariable logistic regression. Odds ratios (ORs) and corresponding 95% confidence intervals with *p* values were estimated. Standard model-building techniques were applied, and the fitness of models was evaluated by using the likelihood ratio test and Akaike's information criterion (AIC), which allowed optimal model improvement and indemnity from multicollinearity among predictors in the model [20]. Forward stepwise algorithm, a stepwise regression approach, was adopted, which started from an intercept and added variables in the model one at a time until the selection criterion (i.e., *p* < 0.05) was reached [21]. All tests were two-sided. The adjusted odds ratios from the multivariable regression were determined, and the strength of association of the independent risk factors/markers with IAV was measured. A map showing town boundaries and IAV-positive cases in Lahore District was digitally produced by using QGIS software (version 3.2, Open Source Geospatial Foundation Project, Boston, MA, USA).

### 2.4. Institutional Review Board Statement

The study was conducted according to the guidelines of the Declaration of Helsinki and approved by the Independent Ethics Committee (IEC) Bioequivalence Study (BeSt) Center, University of Veterinary and Animal Sciences, Lahore, Pakistan (Letter no. IEC – 438, 2016).

### 2.5. Informed Consent Statement

We requested written informed consent from all study participants aged 18 years or older. Surrogate consent was obtained from parents or legal guardians of patients younger than 18 years. Each participant was given a consent form (in local and English language, where appropriate) and verbally briefed about the research objectives and procedures. A well-trained team member registered with Pakistan Medical and Dental Council (PMDC) collected swab samples. The team members were formally trained to collect data during a face-to-face interview.

## 3. Results

As previously reported, a total of 513,126 patients with respiratory signs reported to LGH surveillance hospital. Of these 513,126 cases, 311 suspected ILI and SARI cases were selected on the basis of WHO revised case definitions of ILI and SARI [12]. Of these, 284 were ILI cases, and 27 were SARI cases. Among 50 confirmed cases of IAV, 14 cases were H1N1pdm09, and 15 were H3N2; the remaining 21 cases were untyped (Figure 1). The median age of all patients was 27 years (range: 0.33–80) in our study, while the median age in Pakistan was 22 years in 2016 [22]. Of 50 IAV-positive patients, 64% (32/50) were within the age group of 0–30 years. Among all enrolled individuals, the proportion of female patients was 65% (*n* = 202), while male patients were 35% (*n* = 109). The proportion of IAV-positive male patients was 23% (25/109), and the proportion of IAV-positive female patients was 12.4% (25/202). Gender was significantly different between IAV-positive and IAV-negative cases (*p* < 0.05). About 88% (44/50) of IAV-positive patients reported to the hospital within 7 days of illness.

Univariable analysis identified 8 independent risk factors/markers for IAV with *p* value ≤ 0.25 (a selection criterion for building multivariable model) (Table 1). The use of antibiotics prior to hospital consultation (*p* = 0.010), fever (*p* = 0.022), and gender (*p* = 0.024) showed strong positive association with IAV infection; exposure history to ILI patients within last 7 days (*p* = 0.136), headache (*p* = 0.182), and sneezing (*p* = 0.229) showed weak positive association with IAV infection. Presence of birds at home (e.g., parrots, pigeons) was dropped as a risk factor due to its correlation with another variable, namely, contact with poultry (any bird including backyard birds or poultry in live bird markets, neighbors' poultry birds, etc.) (Spearman rank-order correlation, *ρ* = 0.9). A set of variables comprising clinical characteristics, comorbidities, exposure status, travel history, vaccination status, and treatment history was not studied further due to either having no association (*p* > 0.25) with IAV or the presence of zero cell value in 2 × 2 contingency tables.

**Figure 2:**
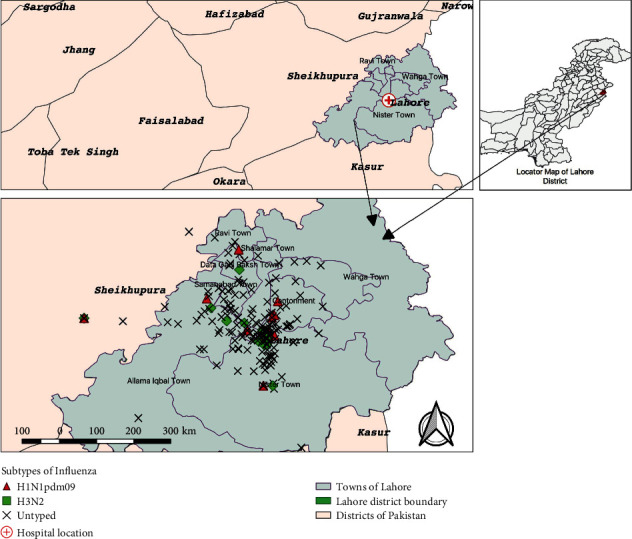
Spatial distribution of influenza A-positive cases with different subtypes from hospital-based sentinel surveillance during Oct 2015–May 2016.

The final multivariable model was established, and three independent variables (headache, sneezing, and contact with poultry) were dropped from the final model due to model-building criteria (*p* > 0.05) during the forward stepwise selection process. The final model included exposure history to ILI patients within last 7 days (OR: 2.09, 95% CI: 1.06–4.13, *p* = 0.033), gender (OR: 2.15, 95% CI: 1.12–4.1, *p* = 0.021), presence of fever (OR: 2.9, 95% CI: 1.38–6.12, *p* = 0.005), and use of antibiotics prior to hospital consultation (OR: 1.89, 95% CI: 0.99–3.63, *p* = 0.054), which were best fitted to the model and were considered to be independent risk factors/markers for influenza A. Among these, fever had the strongest association (*p* = 0.005) with IAV (Table 2). A spot map showed clustering of positive cases of various subtypes of influenza A near the sentinel hospital in Lahore (Figure 2).

## 4. Discussion

Influenza is a major global cause of morbidity and mortality especially in children and in patients with comorbidities [23]. The current study provided population-based estimates of risk factors for IAV infections in a sentinel hospital of Lahore. The results of this study will be a valuable contribution to the available literature about the risk factors associated with influenza-associated illnesses prevalent in low- and middle-income countries. We analyzed a set of risk factors and risk markers for IAV infection, and the results indicated that exposure history to ILI patients within last 7 days, gender, presence of fever, and use of antibiotics prior to hospital consultation are associated with IAV infection.

Our multivariable multiple logistic regression model indicated that history of exposure to the ILI patients within the last seven days is independently associated with a high risk of developing IAV infection. IAV mainly spreads when an infected person coughs and sneezes, transmitting infected droplets to those within 3 feet perimeter. Documented groups of people with high potential for transmission of influenza are household contacts, students, friends, and athletes, with students considered to be “super spreaders.” Most people exposed to a new influenza virus remained asymptomatic; however, serious influenza infection develops in some cases, with likelihood depending on the history of exposure, size of the initial inoculum, or degree of host susceptibility [24, 25]. Our result mirrors the finding of a study conducted in Oman, which showed that exposure to a confirmed case of H1N1 within the last seven days is a significant predictor for H1N1 infection in addition to high-grade fever, sore throat, myalgia, diarrhea, and being of young age [26]. In our study, about 64% of the IAV infected patients were ≤30 years. Another study estimated the secondary attack rate of influenza to be between 10.3% and 20.2% among adult household members and children; this age-related difference in influenza infection might be due to differences in contact behavior associated with children having many contacts in schools or daycare centers, less adherence to cleanliness measures such as hand washing, and weaker immunity levels than adults [27]. Contrary to these studies, a recent comparison between COVID-19 and influenza patients revealed that COVID-19 patients had a more frequent history of household contact with a SARS-CoV-2-infected person compared to influenza patients, who had significant history of frequent travel abroad [28].

We found that being male is significantly associated with IAV infection, although females were overrepresented (65%) in our study. Several studies revealed that males engage in more outdoor activities and have a greater chance of exposure to pathogens, including influenza, via close contact with infected people or contaminated surfaces [29]. Females have better compliance with hygienic measures such as hand washing, which could have reduced the risk of viral infections [30]. Males and females differ in influenza pathogenesis due to sex-based steroid hormones, including estrogen, progesterone, and testosterone, which affect immune responses [29]. Additionally, genes and even microbiota affect immunity to influenza differently in both sexes [31]. Previous studies showed that influenza notifications were predominant during earlier and older life in males and during the premenopausal period in females. Moreover, sex-specific anatomic changes in prepubescent boys often resulted in viral infections due to their shorter upper airways, which could increase their vulnerability to respiratory tract infections [32, 33]. These findings support our results; however, further investigations are needed to determine the effect of sex on influenza pathogenesis.

In our study, 30% (15/50) of IAV-positive patients also reported fever. Among positive cases, almost 88% reported to the hospital within 7 days of onset of symptom. We found that fever is independently associated with a high risk of IAV infection. Febrile response has been a clinical indicator of disease among mammals since ancient times and remains a prominent feature of clinical spectrum, disease pathogenesis, and disease outcome. Previously, it has been reported that the best clinical predicators of influenza are fever and cough, with a positive predictive value of 79% (*p* < 0.001). Hence, patients with ILI having fever and cough had a high probability of testing positive for IAV during peak influenza circulation in the community, making it the right time to initiate influenza antiviral therapy [34]. A systemic review of clinical and epidemiological features of pandemic influenza A (H1N1) 2009 also showed that cough and fever are the most common clinical symptoms and are seen in more than 84% of confirmed influenza cases; thus, these symptoms could be suitable for symptomatic screening [35]. Furthermore, in Thailand, novel risk factors for severe influenza A(H1N1)pdm09 were obesity, pregnancy, bacterial coinfections, and D222/N variants or 225G substitutions of the viral genome. However, the most common clinical presentations were fever and cough, which were not included as risk factors [36]. A recent study compared the clinical presentation of COVID-19 and influenza and reported that all cases of influenza were symptomatic and had a higher frequency of fever, cough, and sore throat, whereas COVID-19 patients more frequently suffered from diarrhea [28]. When comparing results of various influenza related studies, study design is important to be considered, because in surveillance studies, data are obtained mostly from tertiary-care hospitals and intensive care units where patients are already sick at the start of data collection, whereas this is not the case in the patient participating in clinical trials [36]. Our study was based on hospital-based prospective surveillance; therefore, our results could only be comparable to reports with similar study design.

Use of antibiotics prior to hospital consultation was identified as a risk marker for IAV infection. Although there is no direct causal relationship between use of antibiotics prior to hospital consultation and influenza infection, this independent variable could be used as an indicator of influenza infection. A potential explanation of our finding is that those patients, who self-medicated with antibiotics, did so to get partial relief and avoid secondary bacterial infections. Several studies reported that irrational and unwise use of antibiotic treatment is prevailing in health practice in many countries in the absence of confirmed clinical indication; e.g., in Australia, the antibiotic prescribing rate is 11% among patients with influenza and 85% among patients with other acute respiratory tract illnesses despite the fact that antibiotics are not recommended by the Australian National Guidelines for flu treatment because of antibiotic resistance, partial effectiveness, and high prices [37, 38], and in England antibiotics were prescribed to 18% of ILI cases without comorbidities and 28% of cases with comorbidities [39]. In Bangladesh, a hospital-based surveillance study reported that 85% of ILI patients received antibiotics and noted an increasing linear trend between prescribing antibiotics and severity of disease (*p* < 0.001) [40]. A study from Korea depicted significant temporal association between unnecessary antibiotic consumption and influenza activity at primary healthcare facilities, which suggested that reducing influenza cases and discouraging the prescription of antibiotics by doctors might reduce inappropriate antibiotic usage [41]. The issue of inappropriate antibiotic use must be addressed due to development of antimicrobial resistance, as over 20,000 potential drug-resistance genes have been detected during analysis of available bacterial genomes [42].

There are few limitations in our study. First, this study was restricted to risk factors/markers associated with IAV only, and other respiratory pathogens were not studied due to economic and logistic feasibility. Second, selection bias could have occurred in our study because we sampled 5-6 patients daily, which might have biased our findings toward sick patients or young patients who lived closer to the hospital and could easily get to a health facility. Furthermore, our questionnaire contained questions, which collected limited information about patient housing, education, occupations, etc. Comprehensive and detailed longitudinal studies with more exploratory questionnaires are advocated to establish a causal relationship between risk factors and IAV.

## 5. Conclusions

The current study confirmed the presence of IAV infection and identified various determinants of infection in Lahore District. These results could provide the baseline information needed to initiate risk assessment and management of ILI or SARI in the community by modifying the identified risk factors, specifically exposure to persons with respiratory tract illness, being male, being febrile, and usage of antibiotics by patients prior to hospital consultation. Careful evaluation and, where possible, modification of these factors could decrease the risk of IAV infection and may prevent imminent outbreaks of IAV in the community. Our findings are valuable for public health policy makers who are involved in influenza control and pandemic planning. To mitigate the threat of influenza pandemics until a universal vaccine is available, we suggest nonpharmaceutical interventions such as hand hygiene and social distancing, restriction of public gatherings, self-isolation, environmental- and self-cleanliness, travel restrictions, and school closures to control transmission of virus. We strongly recommend easy access to healthcare facilities for every individual of the community for early detection and management of IAV cases to reduce its burden in the community.

## Figures and Tables

**Table 1:** Univariable analysis of potential risk factors/risk markers for IAV with *p* value ≤ 0.25 among ILI and SARI cases during hospital-based surveillance.

Risk factors	Response level	IAV-positive cases (%)	IAV-negative cases (%)	Odds ratio	95% CI	*p* value
Exposure history to ILI patients within the last 7 days	No	31 (13.9)	192 (86.1)	1.71	0.90–3.21	0.136
	Yes	19 (21.6)	69 (78.4)			

Gender	Female	25 (12.4)	177 (87.6)	2.11	1.14–3.88	0.024
	Male	25 (23)	84 (77)			

Birds kept at home	None	45 (15.3)	249 (84.7)	Reference		0.080
	1 = parrots	1 (10)	9 (90)	0.61	0.08–4.97	
	2 = pigeons	2 (66.7)	1 (33.3)	11.07	0.98–124.62	
	3 = others	2 (50)	2 (50)	5.53	0.76–40.30	

Fever	No	35 (13.7)	221 (86.3)	2.37	1.18–4.73	0.022
	Yes	15 (27.3)	40 (72.7)			

Headache	No	24 (13.4)	155 (86.6)	1.58	0.86–2.90	0.182
	Yes	26 (19.7)	106 (80.3)			

Sneezing	No	15 (12.5)	105 (87.5)	1.57	0.81–3.01	0.229
	Yes	35 (18.3)	156 (81.7)			

Use of antibiotics prior to hospital consultation	No	26 (12.2)	187 (87.8)	2.33	1.25–4.32	0.010
	Yes	24 (24.5)	74 (75.5)			

Contact with birds	No	45 (15.3)	249 (84.7)	2.31	0.77–6.86	0.230
	Yes	5 (29.4)	12 (70.6)			

ILI: influenza-like illness; SARI: severe acute respiratory illness.

**Table 2:** Multivariable analysis of risk factors/risk markers for IAV among ILI and SARI cases during hospital-based surveillance.

Risk factors	Response level	Regression coefficient	Standard error	OR	95% CI	*p* value
Exposure history to ILI patients within the last 7 days	No	0.7381	0.3465	2.09	1.06–4.13	0.033
	Yes					

Gender	Female	0.7634	0.3310	2.15	1.12–4.1	0.021
	Male					

Fever	No	1.0658	0.3801	2.9	1.38–6.12	0.005
	Yes					

Use of antibiotics (prior to hospital consultation)	No	0.6389	0.3313	1.89	0.99–3.63	0.054
	Yes					

ILI: influenza-like illness; SARI: severe acute respiratory illness.

## Data Availability

The data gathered and generated during the current study are available from the corresponding author (Mamoona Chaudhry) on reasonable request.

## Supplementary Materials

Supplementary Materials The supplementary file contains the Questionnaire of Sentinel Surveillance of Influenza A Viruses in Lahore District, Pakistan: 2015-2016.
