# Supplementary material for: Risk Factors of Influenza-Associated Respiratory Illnesses Reported to a Sentinel Hospital of Lahore, Pakistan: 2015-2016
Source: Can J Infect Dis Med Microbiol. 2021 Oct 27;2021:2460553. doi: 10.1155/2021/2460553 (PMC8566087; doi:10.1155/2021/2460553)
Supplement: Supplementary Materials — The supplementary file contains the Questionnaire of Sentinel Surveillance of Influenza A Viruses in Lahore District, Pakistan: 2015-2016. [file 2460553.f1.docx]

Sentinel Surveillance of Influenza A Viruses in Lahore District Pakistan: 2015-2016

**Supplementary Material (Questionnaire)**

|  |
| --- |

|  |
| --- |

**Hospital identification no. Date of Interview:**

|  |  |
| --- | --- |

**Patient case no.**

|  |  |
| --- | --- |

**Questionnaire. no**.

**Patient’s Details**

| **Name:** | **Father’s/husband’s name:** |
| --- | --- |
| **Mobile no.** | **Email:** |
| **Address:** | **District:** |

**1. Age in years or date of birth** (yyyy/mm/dd): _____________

**2. Gender**  1. Male  2. Female

**3. Marital Status** 1. Married  2. Unmarried

3. Widow  4. Divorce

**4.Type of Family**  1. Nuclear  2. Extended

**5. Education:**

1. Illiterate  2. Primary

3. Secondary  4. Intermediate

5. Graduate/post graduate

**6. Occupation:**

1. Govt/private employee  2. Jobless grad/businessman

3. House wife  4. Health professional  5. Drivers  6. Others (specify)

**7. Income/Month in rupees**

01. Less than 10000

02. 10000 to 15000

03. 15000 to 20000

04. More than 20000

**8. Date of onset of symptoms ___________________________**

**9. Symptom at any stage of course of infection Yes** **No**

**10. If yes to question no. 9 then specify:**

1. Fever ≥ 38°C/100.4ᵒF  2. History of fever (not measured)

3. Sore throat

4. Shortness of breath or difficulty in breathing

5. Sneezing  6. Cough

7. Running nose  8. Nausea

9. Vomiting  10. Diarrhea

11. Headache  13. Seizures

14. Altered Consciousness  15. Muscle Pain

16. Joint pain  17. Epistaxis/Nose bleeding

18. Conjunctivitis  19. Multiple symptoms

**11.Do you know about influenza vaccine? Yes** **No**

**12. Vaccination with seasonal influenza vaccine within the last year? Yes** **No**

**13. Vaccination with other vaccine? Yes** **No**

**14. If yes then specify (pneumococcal vaccine, swine influenza vaccine)? Yes** **No**

**15. Did the case have any pre-existing condition? Yes** **No**

**16. If yes to question no. 15 then specify**

01. Diabetes  02. Heart Disease  03. Seizure Disorder  04. Lung Disease  05. Asthma  06. Pregnancy (specify months)

07. Obesity  08. Malnutrition

09. Immune deficiency/ HIV  10. Tuberculosis

11. Hepatitis B  12. Hepatitis C

13. Allergy  14. Other diseases

15. Multiple conditions (specify)

**17. Did patient receive antibiotics? Yes** **No**

**18. Did case receive any antiviral therapy? Yes** **No**

**19. If yes to question no. 18 then specify:**

1. Amantadine  2. Remantadine

3. Oseltamivir  4. Zanamivir

**20. Exposure history to ILI patients** (7 day before the onset of illness)? **Yes** **No**

**21. If yes to question no. 20, specify date: __________**

**22. Did you travelled 7 days before the onset of illness? Yes** **No**

**23. Any bird kept at home None  Parrots  Pigeons  Others **

**24. History of contact with poultry? Yes** **No**

**25. Regular use of public transport? Yes** **No**
